# HGPGD: The Human Gene Population Genetic Difference Database

**DOI:** 10.1371/journal.pone.0064150

**Published:** 2013-05-22

**Authors:** Yongshuai Jiang, Ruijie Zhang, Hongchao Lv, Jin Li, Miao Wang, Yiman Chang, Wenhua Lv, Xin Sheng, Jingjing Zhang, Panpan Liu, Jiajia Zheng, Miao Shi, Guiyou Liu

**Affiliations:** 1 College of Bioinformatics Science and Technology, Harbin Medical University, Harbin, China; 2 Graduate University of Chinese Academy of Sciences, Beijing, China; 3 Department of Epidemiology and Statistics, School of Public Health, Central South University, Changsha, China; 4 Tianjin Institute of Industrial Biotechnology, Chinese Academy of Sciences, Tianjin, China; University of North Carolina, United States of America

## Abstract

Demographic events such as migration, and evolutionary events like mutation and recombination, have contributed to the genetic variations that are found in the human genome. During the evolution and differentiation of human populations, different functional genes and pathways (a group of genes that act together to perform specific biological tasks) would have displayed different degrees of genetic diversity or evolutionary conservatism. To query the genetic differences of functional genes or pathways in populations, we have developed the human gene population genetic difference (HGPGD) database. Currently, 11 common population genetic features, 18,158 single human genes, 220 KEGG (Kyoto Encyclopedia of Genes and Genomes) human pathways and 4,639 Gene Ontology (GO) categories (3,269 in biological process; 862 in molecular function; and 508 in cellular component) are available in the HGPGD database. The 11 population genetic features are related mainly to three aspects: allele frequency, linkage disequilibrium pattern, and transferability of tagSNPs. By entering a list of Gene IDs, KEGG pathway IDs or GO category IDs and selecting a population genetic feature, users can search the genetic differences between pairwise HapMap populations. We hope that, when the researchers carry out gene-based, KEGG pathway-based or GO category-based research, they can take full account of the genetic differences between populations. The HGPGD database (V1.0) is available at http://www.bioapp.org/hgpgd.

## Introduction

Any two unrelated individuals share about 99.9% of their genomic DNA sequence. The approximately 0.1% that is different is important in explaining some human phenotypic differences, such as skin color [1], susceptibility to disease and response to pharmacological agents [2], [3], [4]. Genetic differences in, for example, drug-related gene regions [5], the neuregulin 1 gene [6] and the enzyme glucokinase (GCK) gene region [7], in different populations have been reported. Although these studies have been successful in understanding some population differences [8], [9], [10], [11], [12], [13], they have focused on only one or several gene regions. With the development of high-throughput technologies, international projects, such as the HapMap project [14], [15], [16], [17] and the 1000 genome project [18], [19], have been developed. The data from these projects allow genome-wide genetic differences to be investigated. We used the SNP genotype data from HapMap to measure population genetic differences in functional gene regions in the 11 HapMap populations.

Furthermore, because genes often act in groups to perform a specific biological function or cellular process, one or a few genes may not represent the functional status of an entire system [20]. Therefore, tools that can be used to identify and analyze population genetic differences from a system-level have been developed. They include human genome annotation databases such as the KEGG (Kyoto Encyclopedia of Genes and Genomes) pathway database [20], [21], [22], [23] and the Gene Ontology (GO) database [24], [25], [26], [27], that have provided detailed descriptions of gene function and made possible the investigation of population genetic differences from the system or functional levels. Recently, pathway-based methods [28], [29], [30], [31] have been developed to determine whether or not a particular pathway plays an important role in the development of a disease or some other physiological process. Pathway-based methods are powerful tools that can give new insights into various biological phenomena from the system or functional levels. In this study, we used a pathway-based method to measure the population genetic differences based on KEGG pathways and GO categories. The genetic differences that we discovered have been included in the HGPGD database.

## Materials and Methods

### HapMap Populations and SNP Genotype Data used in the HGPGD Database

The genetic differences of single genes, KEGG pathways and GO categories were calculated based on the SNP genotype data in the International HapMap project. Currently, 11 sample populations are in the HapMap database. The 11 populations are: African Americans from the American Southwest (ASW), Utah residents with Northern and Western European ancestry from the Centre d'Étude du Polymorphisme Humain (CEPH) collection (CEU), Han Chinese in Beijing, China (CHB), Chinese in Metropolitan Denver, Colorado (CHD), Gujarati Indians in Houston, Texas (GIH), Japanese in Tokyo, Japan (JPT), Luhya in Webuye, Kenya (LWK), Mexican ancestry in Los Angeles, California (MEX), Maasai in Kinyawa, Kenya (MKK), Toscans in Italy (TSI), and Yoruba in Ibadan, Nigeria (YRI). A sample population of 1,117 unrelated individuals was selected from the HapMap populations [32]. A total of 1,063,592 autosomal SNPs in all 11 sample populations were selected; 987,019 of them passed the quality control (QC). The QC standards are: threshold of 0.001 for the Hardy-Weinberg equilibrium (HWE) test; threshold of 0.75 for the call ratio; and threshold of 0.01 for minor allele frequency (MAF). We calculated the genetic differences of single genes, KEGG pathways and GO categories pairwise between each population pair. The total number of pairwise populations is 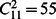.

### Measuring Population Genetic Differences for a Single Gene

The human gene position information was obtained from the ‘‘seq_gene’’ file on the NCBI ftp website. In the HGPGD database, we used 18,158 autosomal genes that contained at least two SNPs in all 11 populations to calculate the population genetic differences. A total of 11 common population genetic features are included in the HGPGD database. The 11 features are mainly related to three genetic aspects: allele frequency, linkage disequilibrium (LD) pattern and transferability of tagSNPs (SNPs in a region of the genome with high LD).

#### Allele frequency

For each gene region, two features are related to allele frequency. (1) MAF (maf): The average differences of allele frequency for each gene region between pairwise HapMap populations were measured. The minor allele in the ASW population was used as the reference. The minor allele frequencies in the ASW population and the frequencies of the same allele in the other populations were calculated. For each gene region, we defined the allele frequency difference 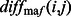 as:
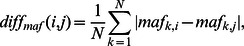
Where 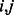 are HapMap populations (1: ASW, 2: CEU, 3: CHB, 4: CHD, 5: GIH, 6: JPT, 7: LWK, 8: MEX, 9: MKK, 10: TSI, 11: YRI); 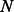 is the SNP counts in a gene region; 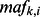 is the frequency of the 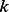
th SNP in population 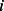; and 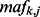 is the frequency of the 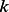
th SNP in population 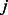. A large 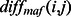 indicates a large difference in MAF in the gene region between population 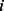 and population 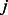. (2) The statistic 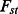, proposed by Weir and Cockerham [33], was calculated for each gene region between pairwise HapMap populations.

#### LD pattern

For each gene region, six features are related to LD pattern. (1) The LD coefficient r^2^ (r^2^): The LD coefficient r^2^ between pairwise SNPs was calculated. (2) D prime (D’): The D’ between between pairwise SNPs was calculated. (3) Block number: The Four Gamete Test [34] was used to identify the haplotype block structure, and the block number within each gene region was calculated. (4) Block size: The average size of blocks within each gene region was calculated. (5) SNP density: The average SNP density of blocks within each gene region was calculated. (6) Haplotype diversity: For each gene region in each block, we calculated the haplotype diversity as: 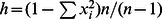, where 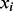 is haplotype frequency and 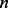 is the sample count [11]. In the present study, we use HaploView v4.1 [35] to complete the identification of each haplotype block. We estimated haplotype frequency using an Expectation Maximization algorithm. LD pattern differences were calculated as: 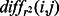, 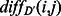, 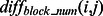, 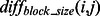, 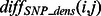 and 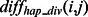 in the same way as 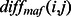.

#### TagSNP transferability

For each gene region, three features are related to tagSNP transferability. (1) Tag percent: The tag percent is the percentage of the number of tagSNPs compared to the total number of SNPs. The tagSNPs were identified using the TAGGER panel in HaploView. The r^2^ threshold was 0.8. (2) Captured percent: For any two populations, A and B, if a SNP in population A exhibited a pairwise r^2^>0.8 with at least one tagSNP selected from population B, then the SNP was defined as a SNP that was captured by population B [11]. The captured percent is defined as the percentage of the number of captured SNPs compared to the total number of SNPs in population A. (3) Average maximum r^2^: The average maximum r^2^ was defined as the average value of the maximum r^2^ between tagSNPs in population A and SNPs captured by these tagSNPs in population B. The differences in tagSNP transferability were calculated as: 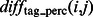, 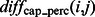 and 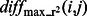 the same way as 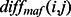.

### Measuring Population Genetic Differences for a KEGG Pathway

Human genes are not independent of each other and genes in the same functional pathway often act together to perform specific biological tasks. Under the action of natural selection, different functional pathways have evolved to display different degrees of genetic differences. The HGPGD database provides the option to query genetic differences in the KEGG pathways in the different HapMap populations.

The KEGG pathways database has been widely used for the systematic analysis of gene functions that involve networks of molecular interactions in cells [20]. In the HGPGD database a total of 220 human functional KEGG pathways are available and each pathway includes at least 10 genes.

The genetic differences for a KEGG pathway were obtained by combining the differences of all the genes in that pathway. In the previous section, we described how the genetic differences of single genes were measured. For pathways, the same weight was assigned to the genes in that pathway and genetic difference scores were calculated separately for each of the 11 features. Genetic difference scores for allele frequency were defined as:
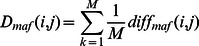
Where 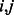 are the HapMap populations (1: ASW, 2: CEU, 3: CHB, 4: CHD, 5: GIH, 6: JPT, 7: LWK, 8: MEX, 9: MKK, 10: TSI, 11: YRI), and 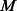
is the gene number in the pathway. 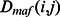 is a measure of the allele frequency difference between population 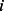
and population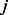. The population differences of the other 10 features were calculated as described above for 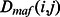.

### Measuring Population Genetic Differences for a GO Category

The GO database provides a controlled vocabulary of terms to define biological descriptors (GO categories) and to support biologically meaningful annotation of gene products [36]. The GO database uses three separate ontologies; biological process (BP), molecular function (MF) and cellular component (CC). In the HGPGD database, there are a total of 4,639 GO categories (BP: 3,269, MF: 862 and CC: 508) all of which have at least 10 genes annotated with those terms. The genes in each GO category were used as a functional gene set to measure genetic differences between HapMap populations.

In the HGPGD database, each GO category is seen as a basic functional unit. The same weight was assigned to genes in the same GO category and the genetic difference scores were calculated. The genetic difference scores for the GO categories were calculated as described above for the KEGG pathways.

## Results

### Overview of the HGPGD Database

The HGPGD database is a freely available database that focuses on population genetic differences in human genes. The current version (v1.0) of the HGPGD database contains 18,158 single human genes, 220 KEGG human pathways and 4,639 GO categories. For each gene, KEGG pathway or GO category, users can obtain the differences in 11 common genetic features between the 11 HapMap populations. Table 1 displays statistical details of the information in the HGPGD database.

**Table 1:** Summary of the data available in the HGPGD database v1.0.

Genetic features	11
Populations	11
Population pairs	55
Genes	18,158
Genetic differences of single gene	10,985,590
KEGG pathways	220
Genetic differences of KEGG pathway	133,100
GO categories (total)	4,639
Genetic differences of GO category (total)	2,806,595
GO categories (biological process, BP)	3,269
Genetic differences of the BP category	1,977,745
GO categories (molecular function, MF)	862
Genetic differences of the MF category	521,510
GO categories (cellular component, CC)	508
Genetic differences of the CC category	307,340

### Selecting a Genetic Feature and Searching the Population Differences for Single Genes

For each of 18,158 single genes in the HGPGD database, by entering a list of Gene IDs, and selecting a population genetic feature, users can obtain the genetic differences between each pairwise HapMap population.

**Figure 1:**
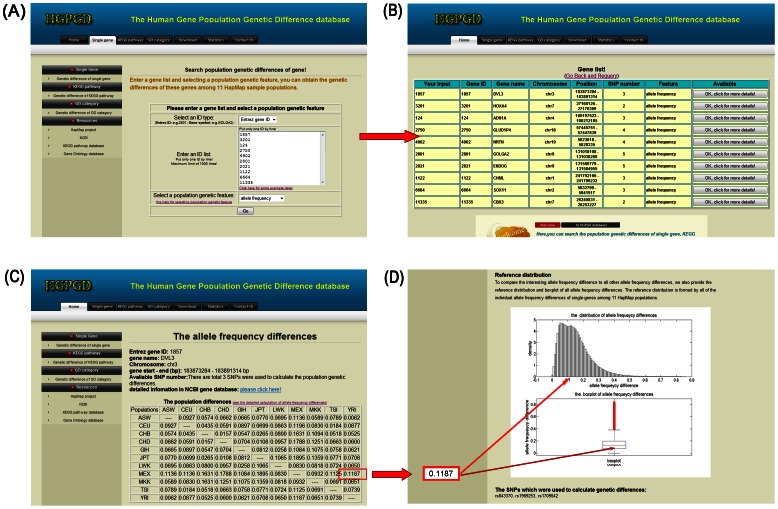
Searching the population differences for single genes. (**A**) Search page. In this example, enter a gene list and select “allele frequency” as the genetic feature. (**B**) Some information about these genes, such as the related gene symbols, chromosome numbers, positions and SNP numbers. (**C**) Detailed genetic differences. A symmetric matrix of allele frequency differences is displayed. Each element in the matrix represents the allele frequency difference between two HapMap populations. (**D**) Reference distribution and boxplot of all the allele frequency differences. The reference distribution and boxplot can be used to compare the allele frequency difference of interest (in this example Gene ID: 1857) to all the other allele frequency differences.

When a list of Entrez Gene IDs is entered and “allele frequency” is selected as the query term (Figure 1A), the search and browse results are displayed in a new page (Figure 1B). As shown (Figure 1B), information about these genes, namely, the related gene symbols, chromosome numbers, positions and SNP numbers are displayed on this page. For more detailed information of genetic differences about these genes, users can click on the link to the detailed information page (Figure 1C). In Figure 1C, a symmetric matrix of allele frequency differences is also displayed. Each row and column of the matrix represents a HapMap population and each element in the matrix represents the allele frequency difference between two populations. To compare the allele frequency difference of interest to all other allele frequency differences, a reference distribution and a boxplot (Figure 1D) of all allele frequency differences are also provided. The reference distribution is the distribution of all of the individual allele frequency differences for the single gene among the 11 HapMap populations.

### Searching the Population Differences for KEGG Pathways

The KEGG pathway search page is similar to the search page for a single gene. When a list of KEGG pathway IDs is entered and a “genetic feature” (e.g. allele frequency) is selected as the query terms, the genetic difference results are displayed on a separate page. The search results include detailed information about the pathway (i.e. pathway name and number of genes in the pathway), and by clicking on the link, the matrix of the genetic differences, reference distribution and boxplot are displayed.

### Searching the Population Differences for GO Categories

The GO category search page is similar to search page for the KEGG pathway. When a list of GO IDs is entered and “genetic feature” (e.g. allele frequency) is selected, the genetic difference results are displayed on a separate page. The search results include detailed information about the GO category (i.e. GO category name and number of genes in the GO category), and by clicking on the link, the matrix of genetic difference, reference distribution and boxplot are displayed.

## Discussion

Human populations have been subjected to a large number of demographic events, such as migration, population expansion and colonization, and, as a result, different populations have been exposed to many different environments. These demographic events together with evolutionary events (such as mutation and selection) have had an effect on the human genome, leading to the population genetic differences that we see today [37], [38]. To be able to easily query the genetic differences between populations, we have developed the HGPGD database. The HGPGD database provides tools to query the genetic differences from a functional perspective. Queries at both the single-gene and system levels are possible via a user-friendly interface. Users can query the HGPGD database by entering either Entrez Gene IDs, gene symbols, KEGG pathway IDs, KEGG pathway names, GO category IDs or GO category names. In future releases of the database, we aim to include genetic differences data for disease genes, GWAS SNPs, drug target genes, microRNA target genes, transcription factor target genes, and BioCarta pathways. Data from the 1000 genome project will also be included. As it grows, the HGPGD database will increasingly become a useful resource that can be mined to obtain a better understanding of the genetic diversity of the various biological components involved in the genetic diversity of human populations.
